# Chemical and Biological Threats, Hazard Potential and Countermeasures

**DOI:** 10.3390/toxics10080444

**Published:** 2022-08-02

**Authors:** Ondrej Soukup, Jan Korabecny

**Affiliations:** 1 Biomedical Research Center, University Hospital Hradec Kralove, Sokolska 581, 50005 Hradec Kralove, Czech Republic; 2 Faculty of Military Health Sciences, University of Defence, Trebesska 1575, 50005 Hradec Kralove, Czech Republic

The scope of this Special Issue is to pay attention to various aspects of toxicology specifically focused on the chemical and biological threats, which may accidentally, or on purpose, endanger human health. Besides the characterization of such threats and their biological consequences, we will focus on the available and novel experimental countermeasures able to provide protection from and/or threaten such exposures. In particular, we have focused, in this Special Issue, on the neuroprotective approaches against organophosphorus poisoning, decontamination approaches against organophosphates and sulphur mustard. From a civilian sphere, we bring original reports that describe the occupational exposure risk to phthalates and diacetyl, commonly occurring plastic components and flavoring additives, respectively, and their health consequences. Finally, we present a review focused on micro and nanoplastics, their relation to human health and their exposure routes through the environment. 

In detail, the team of M. Braga, in a paper on the antiseizure and neuroprotective efficacy of midazolam in comparison with tezampanel (LY293558) against soman-induced status epilepticus, present the neuroprotective efficacy of novel glutamatergic inhibitor tezampanel in comparison to commonly used midazolam (MDZ). The neuroprotective efficacy of the two drugs was studied in the basolateral amygdala, 30 days post-exposure. To highlight the findings, significant neuronal and interneuronal loss, reduced ratio of interneurons to the total number of neurons, and reduction in spontaneous inhibitory postsynaptic currents, accompanied by increased anxiety, were found in the MDZ-treated group. Rats treated with tezampanel did not differ from the control rats (not exposed to soman) in any of these measurements. Thus, tezampanel has significantly greater efficacy than midazolam in protecting against prolonged seizures and brain damage caused by acute nerve agent exposure [1]. Another work, ‘Molecular Evidence on the Inhibitory Potential of Metformin against Chlorpyrifos-Induced Neurotoxicity’ by Daniali et al, presents an in vivo study on the neuroprotective effect of metformin upon chlorpyrifos (CPF) poisoning. Indeed, following the 28 days of CPF and metformin administration, the levels of inflammatory biomarkers, such as tumor necrosis factor alpha (TNFα) and interleukin 1β (IL-1β), as well as the expression of 5HT1 and 5HT2 genes, were analyzed. Moreover, the levels of malondialdehyde (MDA), reactive oxygen species (ROS), and the ADP/ATP ratio, in addition to the activity of acetylcholinesterase (AChE) and superoxide dismutase (SOD), were tested through in vitro experiments. This study demonstrated the potential role of metformin in alleviating the mentioned biomarkers, which can be altered negatively as a result of CPF toxicity. Moreover, metformin showed protective potential in modulating inflammation, as well as oxidative stress, the expression of genes, and histological analysis, in a concentration-dependent manner [2]. Finally, Kassa et al. in their paper ‘Influence of Experimental End Point on the Therapeutic Efficacy of Essential and Additional Antidotes in Organophosphorus Nerve Agent-Intoxicated Mice’ report the effect of antinicotinic compound MB327 on the survival of mice upon nerve agents (sarin, soman, tabun and cyclosarin) exposure. To sum up, MB327 increased the therapeutic efficacy of atropine alone for sarin, soman and tabun intoxication, and that of the standard antidotal treatment (atropine and oxime) for sarin and tabun intoxication; however, the therapeutic efficacy of MB327 was lower than the oxime-based antidotal treatment. To compare the 6 and 24 h end points, the influence of the experimental end point was not observed, with the exception of the higher dose of MB327. Despite the fact that only a negligible beneficial impact of the compound MB327 was observed, antinicotinics may offer an additional avenue for countering poisoning by nerve agents that are difficult to treat. Of note, LD_50_ values of sarin, soman, tabun and cyclosarin, both treated and untreated, in mice for experimental end points of 6 and 24 h are reported in this study [3].

The second area covered by this Special Issue deals with the decontamination of chemical warfare agents. Markova et al. in their study ‘**Synthesis and Decontamination Effect on Chemical and Biological Agents of Benzoxonium-Like Salts**’ describe antimicrobial, as well as decontamination, potential of novel series based on benzoxonium scaffolds. In particular, biocidal activity against a panel of bacterial strains, including *Staphylococcus aureus* in biofilm form and *Francisella tularensis* as a representative of potential biological warfare agents, was screened. From a point of view of decontamination potential, the efficiency of BOC-like compounds to degrade the organophosphate simulant fenitrothion was examined. In summary, despite the fact that no single compound with universal effectiveness was identified, a mixture of only two compounds from this group would be able to satisfactorily cover the proposed decontamination spectrum. Furthermore, the dual effect on chemical and biological agents of benzoxonium-like salts offer attractive potential as active components of decontamination mixtures in the case of a terrorist threat or chemical or biological accidents [4]. In another work entitled ‘**Reactive Organic Suspensions Comprising ZnO, TiO2, and Zeolite Nanosized Adsorbents: Evaluation of Decontamination Efficiency on Soman and Sulfur Mustard**’ by Ginghina and Bratu, the decontamination efficiency of three types of reactive organic suspensions (based on nanosized adsorbents) on two real chemical warfare agents, soman (GD) and sulfur mustard (HD), is described. Three types of nanoparticles (ZnO, TiO2, and zeolite) were employed in the decontamination formulations for enhancing the degradation of the toxic agents. The conversion of the two chemical warfare agents into their decontamination products was also monitored up to 24 h. Four main degradation products, resulting from the decontamination of sulfur mustard, and five main degradation products, resulting from the decontamination of GD, were identified and quantified by the GC-MS technique as well. In terms of efficacy, the organic suspensions that comprised ZnO, TiO2, and zeolite nanoparticles proved their decontamination efficiency on soman and sulfur mustard, whereas the conversion study into the harmless degradation products offers a comprehensive image on the decontamination process [5].

Finally, the third part that refers to the occupational exposures is introduced by a review entitled ‘Scientific Evidence about the Risks of Micro and Nanoplastics (MNPLs) to Human Health and Their Exposure Routes through the Environment’ by Rodrigues et al., focusing mainly on ingestion and inhalation routes and their medical consequences [6]. Inhalation route of exposure is the subject of an original work entitled ‘Diacetyl Vapor Inhalation Induces Mixed, Granulocytic Lung Inflammation with Increased CD4^+^CD25^+^ T Cells in the Rat’ by McGraw’s group. Diacetyl (DA) is a highly reactive alpha diketone associated with flavoring-related lung disease. The study characterizes different T cell populations within the lung following repetitive DA vapor exposures. In particular, while no significant change was observed in percent lung CD3^+^, CD4^+^, or CD8^+^ T cells, a significant increase in lung CD4^+^CD25^+^ T cells developed after 1 week that persisted at 2 weeks post-exposure. In addition, BALF IL-17a increased significantly after 2 weeks in DA-exposed rats compared to the air controls. Lung CD4^+^CD25^+^ T cells and BALF IL17a correlated directly with BALF total protein and inversely with rat oxygen saturations. In summary, repetitive DA vapor exposure at occupationally relevant concentrations induced mixed, granulocytic lung inflammation with increased CD4^+^CD25^+^ T cells in rats [7]. Finally, the original paper ‘Risk of Abdominal Obesity Associated with Phthalate Exposure of Nurses’ by Kolena et al assesses potential phthalate exposure among nurses by high-performance liquid chromatography and tandem mass spectrometry and anthropometric measurements, along with questionnaires. As a result, associations between mono-benzyl phthalate (MBzP) and body mass index (BMI), hip circumference (HC), waist circumference (WC), waist to height ratio (WHtR), and fat mass index (FMI), visceral fat content, BMI risk and hip index risk (HIrisk) were observed, suggesting that occupational exposure to phthalates may induce abdominal obesity and result in obesity-related metabolic disorders [8].

To conclude, this Special Issue describes important findings related to chemical and biological threats, their hazard potential on human health and potential countermeasures. All these findings broaden the knowledge in this field and will stimulate further research, which hopefully will result in an impact on practical applications in the near future. 
